# Beneficial Effects of the Activation of the Angiotensin-(1–7) Mas Receptor in a Murine Model of Adriamycin-Induced Nephropathy

**DOI:** 10.1371/journal.pone.0066082

**Published:** 2013-06-07

**Authors:** Kátia Daniela Silveira, Lívia Corrêa Barroso, Angélica Thomáz Vieira, Daniel Cisalpino, Cristiano Xavier Lima, Michael Bader, Rosa Maria Esteves Arantes, Robson Augusto Souza dos Santos, Ana Cristina Simões-e-Silva, Mauro Martins Teixeira

**Affiliations:** 1 Imunofarmacologia, Departamento de Bioquímica e Imunologia, Universidade Federal de Minas Gerais, Belo Horizonte, Minas Gerais, Brazil; 2 Departamento de Patologia Geral, Universidade Federal de Minas Gerais, Belo Horizonte, Minas Gerais, Brazil; 3 Departamento de Pediatria da Faculdade de Medicina, Universidade Federal de Minas Gerais, Belo Horizonte, Minas Gerais, Brazil; 4 Departamento de Fisiologia e Biofísica, Universidade Federal de Minas Gerais, Belo Horizonte, Minas Gerais, Brazil; 5 Max Delbrück Center for Molecular Medicin, Berlin Buch, Germany; INSERM, France

## Abstract

Angiotensin-(1–7) [Ang-(1–7)] is a biologically active heptapeptide that may counterbalance the physiological actions of angiotensin II (Ang II) within the renin-angiotensin system (RAS). Here, we evaluated whether activation of the Mas receptor with the oral agonist, AVE 0991, would have renoprotective effects in a model of adriamycin (ADR)-induced nephropathy. We also evaluated whether the Mas receptor contributed for the protective effects of treatment with AT_1_ receptor blockers. ADR (10 mg/kg) induced significant renal injury and dysfunction that was maximal at day 14 after injection. Treatment with the Mas receptor agonist AVE 0991 improved renal function parameters, reduced urinary protein loss and attenuated histological changes. Renoprotection was associated with reduction in urinary levels of TGF-β. Similar renoprotection was observed after treatment with the AT_1_ receptor antagonist, Losartan. AT_1_ and Mas receptor mRNA levels dropped after ADR administration and treatment with losartan reestablished the expression of Mas receptor and increased the expression of ACE2. ADR-induced nephropathy was similar in wild type (*Mas^+/+^*) and Mas knockout (*Mas* ^−/−^) mice, suggesting there was no endogenous role for Mas receptor activation. However, treatment with Losartan was able to reduce renal injury only in *Mas^+/+^*, but not in *Mas* ^−/−^ mice. Therefore, these findings suggest that exogenous activation of the Mas receptor protects from ADR-induced nephropathy and contributes to the beneficial effects of AT_1_ receptor blockade. Medications which target specifically the ACE2/Ang-(1–7)/Mas axis may offer new therapeutic opportunities to treat human nephropathies.

## Introduction

Angiotensin-(1–7) [Ang-(1–7)] is a biologically active heptapeptide that has been postulated to counterbalance the physiological actions of angiotensin II (Ang II) within the renin-angiotensin system (RAS) [1]. Ang-(1–7) was initially regarded as an inactive component of the RAS for many years [2]. However, in recent years, several key findings have increased our understanding of the RAS and the biological significance of Ang-(1–7). This peptide is present in the circulation and in many tissues, including heart, vessels and kidney [2].

Numerous experimental and clinical studies have shown that the inhibition of the ACE-Ang II-AT_1_ receptor axis reduces renal dysfunction and fibrosis [3]–[5]. Therefore, ACE inhibitors (ACEi) and AT_1_ receptor blockers (ARBs) have been used as first-line therapies to reduce the progression of chronic kidney diseases (CKD) [3], [6]. Accumulating evidence suggests that, in addition to Ang II, Ang-(1–7) also plays a key role in regulating renal function by acting at glomerular and tubular sites [7]–[9]. Ang-(1–7) increased renal blood flow in anesthetized rats, modulated sodium and water excretion [10], [11], reduced urinary protein excretion [12], [13], partially restored renal vascular responsiveness and produced renal vasodilatation in diabetic SHR rats [14]. The renal effects of Ang-(1–7) were mimicked by the synthetic oral agonist of the Mas receptor, the compound AVE 0991 in the kidney [7] as well in other disease models [15].

On the other hand, there still have been controversial findings concerning the pathophysiological role of Ang-(1–7) in the context of normal renal function or during disease states [16], [17]. In this regard, while Pinheiro et al [16] showed that genetic deletion of Mas receptor in C57BL/6 mice led to glomerular hyperfiltration, proteinuria and renal fibrosis, Esteban et al [17] reported that renal deficiency for Mas diminished renal damage in unilateral ureteral obstruction and ischemia/reperfusion injury and the infusion of Ang-(1–7) to wild-type mice elicited inflammatory response. So far, whether the actions of Ang-(1–7) on renal function do indeed counter act those of Ang II in the context of disease states remains to be shown.

In this context, the present study aimed to investigate the capacity of Mas receptor activation to protect against renal damage by using the classical model of Adriamycin (ADR)-induced nephropathy [18], [19]. Adriamycin injection mimics several features of human nephrotic syndrome and is characterized by interstitial and glomerular infiltration of leukocytes, fibrosis, and proteinuria [18], [19]. By using this model, we evaluated whether Mas receptor activation with the oral agonist, AVE 0991, would have renoprotective effects. We also evaluated whether the Mas receptor was relevant for the well-known renoprotection elicited by treatment with AT_1_ receptor blockers [3], [4].

## Materials and Methods

### Animals

In the present study, we used Balb/c, FVBN wild-type (Mas^+/+^) or FVBN Mas receptor knockout (*Mas* ^−/−^) mice. All mice were male and 8–10 weeks old. Mice were maintained under temperature-controlled conditions with an artificial 12-h light/dark cycle and were fed standard chow and water *ad libitum*. Animals were bred at the animal facility of the Universidade Federal de Minas Gerais (UFMG) and the study was approved by the local animals Ethics Committee.

### Experimental design

A single dose of ADR (10 mg/kg) (adriablastina rd, Pfizer, SP, Brazil) [19] was injected in the tail vein of non-anesthetized mice. Animals developed proteinuria at the seventh day after injection and were evaluated by three weeks (21 days).

The first set of experiments aimed to investigate the effect of Mas receptor activation on renal function and histology. These effects were also compared to those elicited by the AT_1_ receptor blocker, Losartan, in the same experimental conditions. Therefore, we used Balb/c mice randomly divided into 4 experimental groups: 1) Sham group - animals receiving a single injection of saline (0.9% NaCl) in the tail vein; 2) Vehicle-treated group - ADR-injected mice that orally received vehicle (tap water), on daily basis, from day 7 to day 14 after ADR injection; 3) AVE-treated group - ADR-injected mice orally treated with 3 mg/Kg of the Mas receptor agonist, AVE 0991 (Aventis Pharma Deutschland, Frankfurt, Germany), on daily basis, from day 7 to day 14 after ADR injection; 4) Losartan-treated group - ADR-injected mice that orally received 10 mg/kg of Losartan (Merck Research Laboratories, Rahway, NJ), on daily basis, from day 7 to day 14 after ADR injection.

The second set of experiments intended to evaluate the putative role of Mas receptor in contributing to the renoprotective effects of AT_1_ receptor blockers upon renal dysfunction and tissue damage. In this set of experiments, we used FVBN wild-type (*Mas^+/+^*) or FVBN Mas receptor knockout (*Mas* ^−/−^) mice that received a single injection of ADR (10 mg/kg) and daily oral doses of AVE 0991 (3 mg/kg) or of Losartan (10 mg/kg), from 7^th^ to 14^th^ day after ADR injection.

### General measurements and renal function parameters

To evaluate the effects of ADR-induced nephropathy, as well as of the treatment with AVE 0991 or Losartan, on renal physiology, some parameters were evaluated. First, mice were housed individually in metabolic cages (Tecniplast, Italy), three days before ADR or saline injection. Groups of animals (n = 6 to 10) were sacrificed at 0, 7, 14 and 21 days following ADR or saline injection. After an adaptation period of three days, urine volume was measured for the next 24 hours. At the end, 24-hours urine samples were collected and centrifuged at 3,000 g for 5 min. Urine was used to determine microalbuminuria and creatinine concentrations. At the same time-point of urine sampling, blood samples were collected from the lower abdominal cava vein, under ketamine and xilazyne anesthesia (150 mg/kg and 10 mg/kg, respectively), and centrifuged at 2,000×g for 15 min at 4°C. The resulting plasma samples were used to measure creatinine and albumin concentrations. Samples of urine and plasma were stored at −20°C until renal function parameters evaluations.

Creatinine and albumin concentrations were determined by enzymatic kit (Bioclin/Quibasa, Belo Horizonte, MG, Brazil) and microalbuminuria was determined by immunoassay (Bioclin/Quibasa, Belo Horizonte, MG, Brazil).

### Systolic blood pressure

In order to evaluate possible changes in blood pressure during ADR-induced nephropathy, systolic blood pressure (SBP) was measured by the tail-cuff method using an XBP1000 series rat tail blood pressure system (Kent Scientific, Torrington, CT), as described previously [20]. Measurements were performed on the three days before ADR injections, as an adaptation of the mice at experimental conditions imposed by equipment. Subsequent measurements were performed, daily, in the 1^st^, 4^th^, 6^th^, 7^th^, 14^th^ and 21^st^ days. Results were reported in mmHg.

### Renal histopathology

Paraffin-embedded sections (4-mm thick) were deparaffinized with xylene and rehydrated through a descending ethanol gradient. Histological sections were examined following periodic acid–Schiff staining, and graded according to published standards [21].

The degree of nephron injury and glomerular fibrosis was assessed by computer-aided image analysis of PAS-stained kidney sections. Under a light microscope (Olympus BX51, Japan) field-images were captured with a digital camera (Megacybernetics) connected to the microscope. A semiquantitative score (glomerular and tubular injury index) was used to evaluate the degree of scarring as described previously [21]. Twenty high-power fields of renal cortex were randomly selected for assessing tubular alterations (atrophy, casts, and vacuolization) and interstitial changes (fibrosis and inflammation) and graded from 0 to 5. Tubulointerstitial area in the cortex was graded as follows: 0, normal; 1, area of interstitial inflammation and fibrosis, tubular atrophy, and vacuolization involving 0–10%; 2, lesion area between 10–20%; 3, lesion area between 20–30%; 4, lesion area between 30–40%; and 5, lesions involving 40–100% of the field). Fifty randomly selected glomeruli were assessed for glomerular damage (well-developed exudative, mesangial proliferation and glomeruli hypertrophy), and graded as follows: 0, normal; 1, slight glomerular damage of the mesangial matrix and/or hyalinosis with focal adhesion involving 10% of the glomerulus; 2, sclerosis of 10–20%; 3, sclerosis of 20–30%; 4, sclerosis of 30–40%; and 5, sclerosis of 40–100% of the glomerulus. All scoring was performed in a blinded manner. The damage was scored semiquantitatively on a scale of 1 to 5.

### Urinary *TGF-β_1_*

Since *TGF-β_1_* has been considered a potential biomarker of renal tissue fibrosis [22], this cytokine was measured in 24-hour urine samples after ADR or saline injection. Levels of *TGF-β_1_* in the urine were assessed by ELISA in accordance with the procedures supplied by the manufacturer (R&D Systems, Minneapolis, MN). Sample of the urine were collected in metabolic cages and stored at −20°C. Until refrigeration, 10 µL of commercial protease inhibitor cocktail (Sigma Aldrich, Saint Louis, USA) were added at urine sample. Results were expressed as relative units of cytokine per mg of urinary creatinine.

### Renal mRNA levels of angiotensin receptors, AT_1_ and Mas, and ACE2

Renal mRNA levels for AT_1_, Mas receptors and ACE2 were estimated by quantitative real time PCR (polymerase chain reaction). First, total RNA was extracted from kidneys using TRIzol® reagent according to the manufacturer's protocol. Reverse transcription was performed using 2 µg of total RNA, 200 U of reverse transcriptase, RT buffer 5X (2.5 µl), 10 mM dNTPs (1.8 µl), RNAsin 10000 U (0.2 µl) and oligo dT 15 50 µM (1.0 µl). The profile of temperatures for this reaction was: 70°C for 5 min then ice for 2 min, then back to the thermocicler for 42°C for 60 min, 70°C for 15 min and 4°C as the final step. Resultant cDNA was used for real time PCR as below. Specific primers were designed using Primer Express software and synthesized by IDT. AT_1_ primer set picks up both AT_1_a andAT_1_b receptor subtypes. Real time PCR was carried out on a StepOne sequence detection system (Applied Biosystems) using SYBR Green PCR Master Mix (Applied Biosystems). The relative levels of gene expression were determined by the comparative threshold cycle method as described by the manufacturer, in which data for each sample is normalized to 18S expression.

### Statistical analysis

A statistical analysis was performed by GraphPad Prism software, release 4.0 (GraphPad Software, San Diego, CA). All data had normal distribution according to the Shaphiro test. Results are expressed as the mean ± SEM. Differences between groups were evaluated by ANOVA, followed by a Student–Newman–Keuls test. The level of significance was set at p<0.05.

## Results

### Time-course of the renal changes following ADR-induced nephropathy

All experimental animals injected with ADR developed nephropathy characterized by proteinuria, hypoalbuminemia, and progressive renal injury. As shown in Table 1, injection of ADR resulted in reduction in body weight that was clear at day 7 day and persisted till day 21. Systolic blood pressure raised progressively from day 7 to day 14 and was still elevated at day 21 as compared to the control group (day 0). Microalbuminuria was detectable at day 7 and was 45-fold greater than baseline at day 21 after disease induction. Loss of albumin in urine was associated with marked fall of serum albumin at all time points evaluated (Table 1). There was no change in serum creatinine throughout the observation period, but urinary creatinine was below control values at days 14 and 21 after ADR injection (Table 1).

**Table 1:** Time course of adriamycin-induced renal dysfunction.

Days	Weight (g)	Systolic Blood Pressure (mmHg)	Microalbuminuria U P/C	Serum Albumin (µg/ml)	Urinary Creatinine (µg/ml)	Plasma Creatinine (µg/ml)
**0**	25.5±0.7	82.4±3.7	0.008±0.004	2.4±0.08	820.4±63.3	2.4±0.5
**7**	20.2±0.8*^#^	93.3±2.1*	0.18±0.03* ^#^	1.5±0.2* ^#^	704.3±75.5	2.8±0.4
**14**	22.9±0.9*	99.8±3.6*^#^	0.36±0.05* ^#^	1.8±0.06*^#^	464.1±80.3*^#^	2.6±0.4
**21**	22.2±1.7*	91.1±3.5*	0.37±0.09*	1.9±0.05 *	534.8±36.3*	2.8±0.3

Adriamycin (10 mg/kg) was injected in the tail vein of Balb/c mice. The following parameters were evaluated at days 7, 14 and 21 after ADR injection: mean body weight (g), systolic blood pressure (mmHg), albumin/creatinine ratio, serum albumin (g/dL), urinary and serum creatinine (mg/dl). Results are mean ± SEM of 6–10 mice per group. (*) for P<0.01 when compared to day 0.

### Histopathologic and scoring evaluation in ADR-induced nephropathy

**Figure 1:**
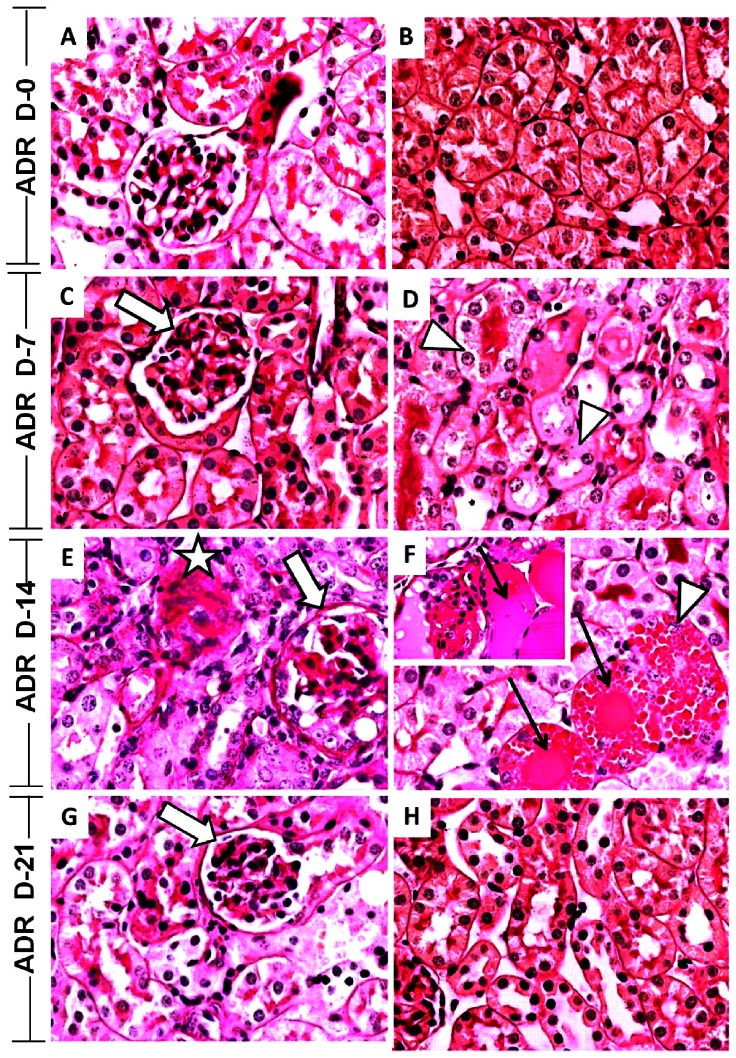
Adriamycin-induced morphological changes in glomerular and tubular regions of the kidney. Representative photographs of PAS-stained glomerular and tubular regions noticed in control mice (Sham, A, B), and 7 (C, D), 14 (E, F) and 21 (G, H) days after injection of adriamycin (10 mg/kg). Glomerular damage (large arrows), tubule-interstitial changes, atrophy of tubular epithelial cells (arrowheads) and tubular enlargement (thin arrows, insert) increased in the renal cortex from day 7 (D) to day 14 (F, insert, arrow). Global sclerosis was observed in many glomeruli (E, asterisks). Resorption droplets were present in tubular cells at day 14 (panel F, thin arrows). Histological changes stabilized on day 21 (G, H). Original magnification 40× objective.

Adriamycin induced both glomerular and tubule-interstitial changes. The severity of changes increased progressively in the renal cortex from day 7 to day 14 and stabilized on 21 (Figure 1A–H). ADR induced nephropathy probably by direct toxic damage to the glomerulus with subsequent tubule-interstitial injury in contrast to the normal glomerular and tubular aspects noticed in sham-operated mice (Figure 1 A, B). At day 7, there was discrete glomerular changes, including segmental fibrin deposition (Figure 1C, arrow), discrete tubular cell damage (figure 1D, arrowheads), expansion of interstitial space and discrete enlargement of tubules. At day 21, focal segmental sclerosis (Figure 1G, arrow) was detected in a small percentage of glomerulus and there was tubular regeneration (Figure 1H, arrowheads).

At day 14 following ADR administration, significant glomerular and tubular injury associated with interstitial inflammation was observed, as shown in Figure 1E and F. There was glomerular enlargement due to significant increase in mesangial matrix area and increased percentage of sclerotic glomeruli (range 40 to 100%), accompanyied by variable degrees of mesangial expansion, increased mesangial cellularity, segmentation, capillary obliteration and formation of cell bridges between the tuft and Bowman's capsule (Figure 1E, arrow). In addition, global sclerosis was observed in many glomeruli (Figure 1E, asterisks). Tubules displayed severe changes, including decrease in height of tubular epithelial cells (tubular atrophy) and vacuolization. Intra-tubular eosinophilic large cast formation (Figure 1F and inset, thin arrows) and focal increase in reabsorption droplets in tubular cells were also observed (Figure 1F, arrowheads). The interstitial volume increased mildly and focally and there was a discrete infiltration of mononuclear cells. Less frequently, glomeruli with minimal lesions embedded in normal tubules could be found adjacent to severely damaged areas, indicating the focal nature of the disease process.

The degree of glomerular and tubular injury was graded as mild injury at day 7, changes were maximum at day 14 and tended to attenuate at day 21 following ADR administration. By 21 days, some glomeruli were reduced in size with several vacuoles, collapse and segmentation of tuft, but mostly the damage scores were reduced. As disease was maximal at day 14, this time point was chosen for subsequent experiments. Scores for glomerular and tubule-interstitial damage averaged 4.2 and 4.6, respectively, at 14 days after ADR administration. These values were significantly higher than those in Sham-operated animals (Glomerular damage, Sham, 0.24±0.04 *vs ADR*, 4.2±0.37; Tubule-interstitial damage, Sham, 0.0±0.0 *vs* ADR, 4.6±0.25, respectively).

### Renal effects of AVE0991 or Losartan administration in ADR-induced nephropathy

**Figure 2:**
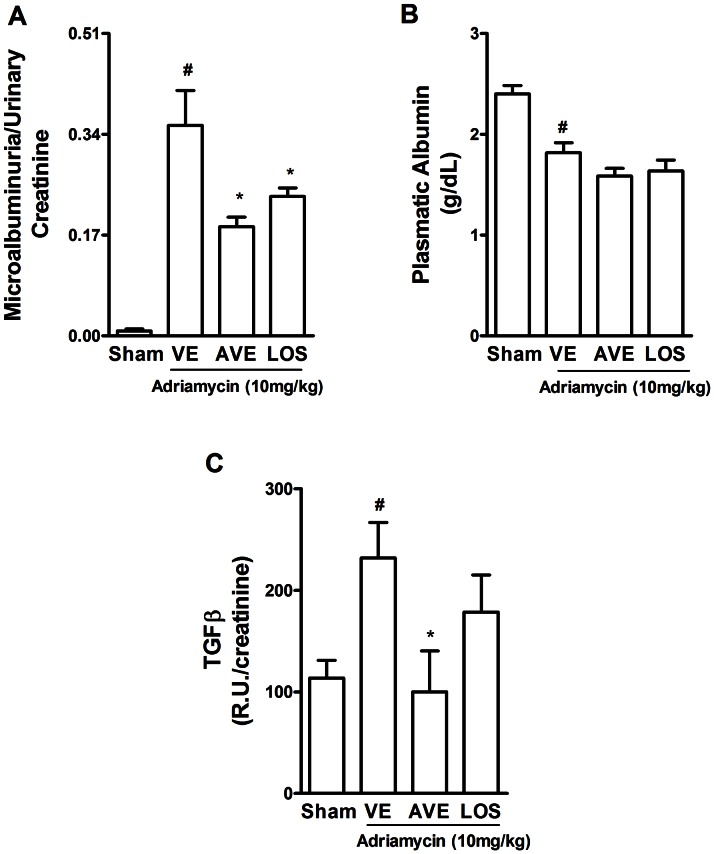
Effects of the treatment with the Mas receptor agonist, AVE 0991, and the AT1 receptor blocker, Losartan, on adriamycin-induced renal injury. Adriamycin (ADR, 10 mg/kg) was injected in the tail vein of Balb/c mice. Animals were treated daily with vehicle (VE, filtered water), AVE0991 (AVE, 3 mg/kg) or Losartan (LOS, 10 mg/kg) by gavage from days 7 to day 14 day after ADR injection. The sham group received a single injection of NaCl 0.9% in the tail vein and was treated with filtered water. Microalbuminuria (A) and serum albumin levels (B) were evaluated on day 14 in n = 6–10 mice per group. The panel also shows urinary levels of TGF-β (C) in n = 4–5 mice per group. (*) for P<0.05 when compared to VE group and (#) for P<0.05 when compared to sham group.

Mice were treated with the Mas receptor agonist, AVE 0991 (3 mg/kg), from day 7 to day 14, as an attempt to mimic the real clinical situation, ie. patients arriving with some degree of injury, but not full blown renal damage. As shown in Figure 2, treatment with AVE 0991 had significant beneficial effects on ADR-induced renal dysfunction and injury. Parallel experiments were carried out with the AT_1_ receptor antagonist, Losartan, which had similar protective effects to those of AVE 0991. Indeed, renal excretion of albumin was significantly reduced by treatment with AVE 0991 (51% decreased, p<0.01, n = 6) or Losartan (34%, p<0.05, n = 6) (Figure 2A). Although injection of ADR induced a decrease in serum levels of albumin (see Table 1), there was no reversion of this parameter by the treatments with the compounds, AVE or Losartan (Figure 2B). Urinary levels of TGF-β were increased at day 14 in mice given adriamycin. Treatment with AVE 0991 also reduced urinary levels of TGF-β1 (Figure 2C).

**Figure 3:**
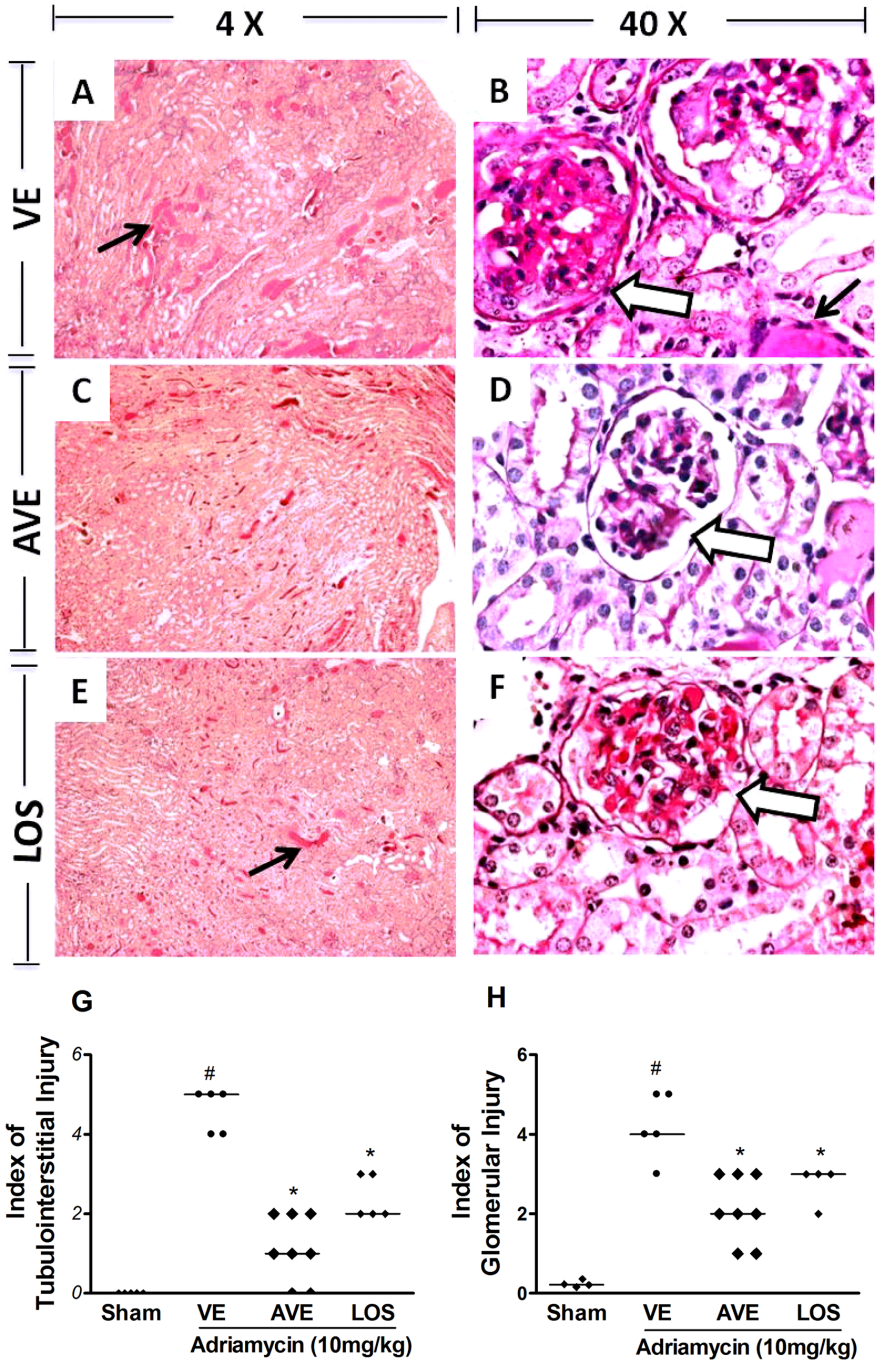
Histological changes and index of tubulointertitial and glomerular injury of ADR-induced nephrosis at day 14. Adriamycin (10 mg/kg) was injected in tail vein of Balb/c mice, on day 0 in vehicle-treated, VE (filter water), AVE0991-treated, AVE, (AVE 0991, 3 mg/kg) and Losartan-treated, LOS groups (losartan, 10 mg/kg). Representative photographs of PAS stained of glomerular and tubular regions were obtained at 14^th^ day. All mice were treated by gavage, daily, 7^th^ to 14^th^ day after ADR-induction. Glomerular (large arrow) and tubular injuries (thin arrows) observed in vehicle-treated mice (A–B) were attenuated by treatment with AVE 0991 (C–D) or Losartan (E–F). Original magnification 4× objective (A, C) and 40× (B, D). The indexes of tubulointerstitial (G) and glomerular (H) injuries were graded in a blind manner, as described in methods section. Symbols represent results in single animals and the trace is median value for 5–8 animals. (*) for P<0.05 when compared to 14^th^ day after ADR-induction group and (#) for P<0.05 when compared to sham group.

Figure 3 shows some of the major changes in the renal architecture of mice given adriamycin and that were subsequently treated with vehicle (Figure 3A–B), AVE 0991 (Figure 3C–D) or Losartan (Figure 3E–F). Glomerular and tubular injuries observed in vehicle-treated mice were attenuated by treatment with AVE 0991 or Losartan, as it can be seen by the semi-quantitative analysis (Figure 3G–H). In both treatments, there was reduction in the intensity of mesangial expansion, mesangial cellularity, adhesion formation (open arrows) and number of glomeruli which were affected, as shown by the arrows. Similar protection occurred in the renal tubules with reduction of focal areas of proteinaceous casts (thin arrows).

### Role of ACE2/Ang-(1–7)/Mas receptor activation in ADR-induced nephropathy and potential interactions with AT_1_ antagonists

**Figure 4:**
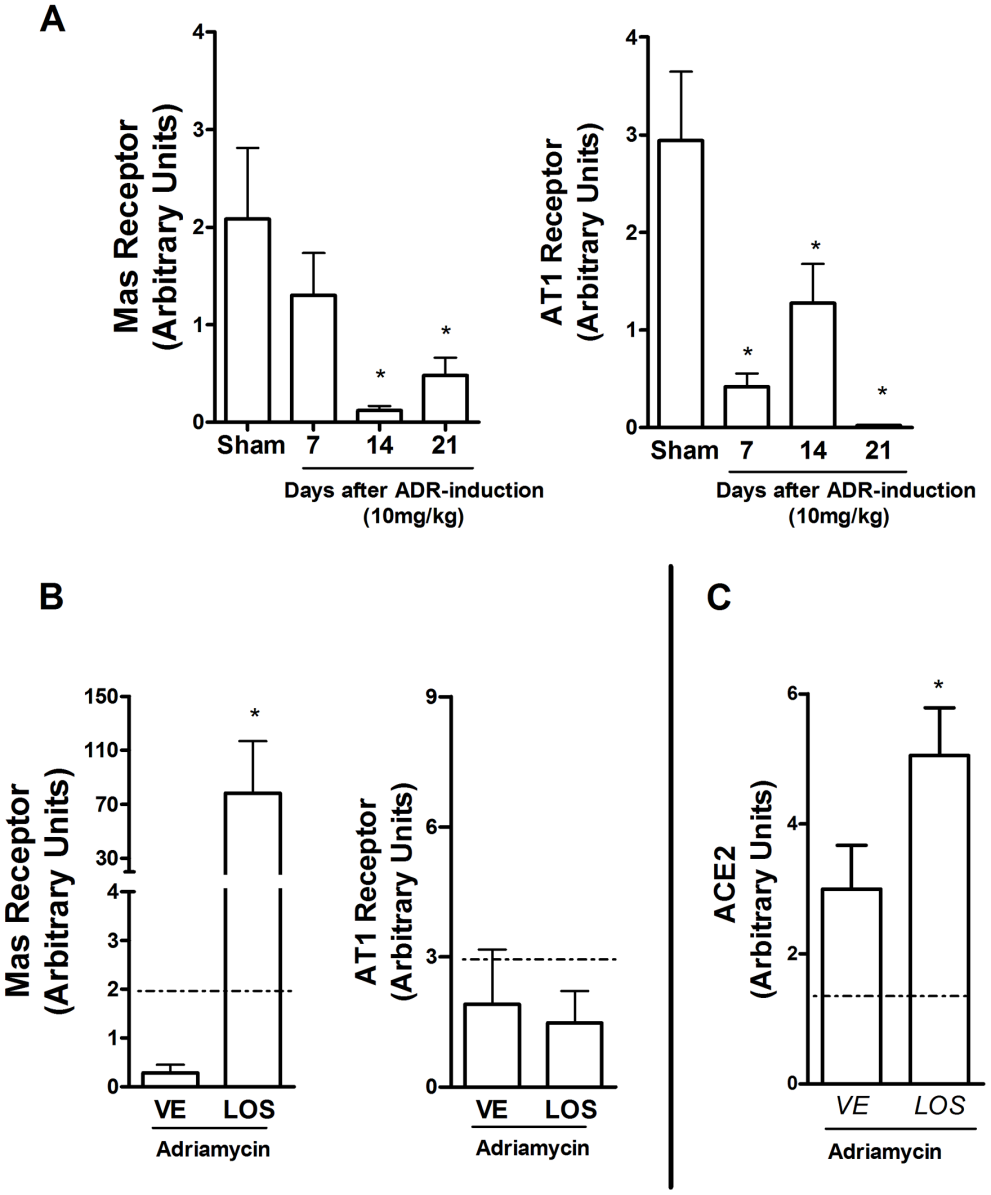
mRNA expression of *Mas*, *AT1* and ACE2 in the kidneys after injection of adriamycin. In A, *Mas* and *AT1* mRNA levels were evaluated in the kidney before (Sham) and 7, 14 and 21 days after injection of adriamycin (ADR, 10 mg/kg). In B, *Mas* and *AT1* mRNA levels at day 14 in kidneys of mice injected with adriamycin that were treated with vehicle (VE) or losartan (LOS, 10 mg/kg). In C, ACE2 mRNA levels at day 14 in kidneys of mice injected with adriamycin that were treated with vehicle (VE) or losartan (LOS, 10 mg/kg). The dotted line across the graphs represents levels in control animals. Renal mRNA levels of receptors and ACE2 were estimated by real time PCR. Results are mean ± SEM of n = 5 mice per group. (*) for P<0.05 when compared to sham group.

Expression levels of angiotensin receptors, AT_1_ and Mas, and ACE2 were evaluated by real time PCR. As shown in Figure 4A, there was marked reduction of expression of both receptors in the kidney after the administration of ADR (Figure 4A). Mas receptor mRNA expression was mostly decreased at day 14, whereas levels of AT_1_ mRNA were lowest at day 21. Treatment with Losartan greatly increased by about 280-fold the expression of Mas receptor mRNA in the kidneys of animals given adriamycin (10 mg/kg) (Figure 4B). Losartan treatment did not alter the levels of AT_1_ receptor mRNA (Figure 4B). Renal expression of ACE2 mRNA was significantly increased following Losartan treatment (Figure 4C).

**Figure 5:**
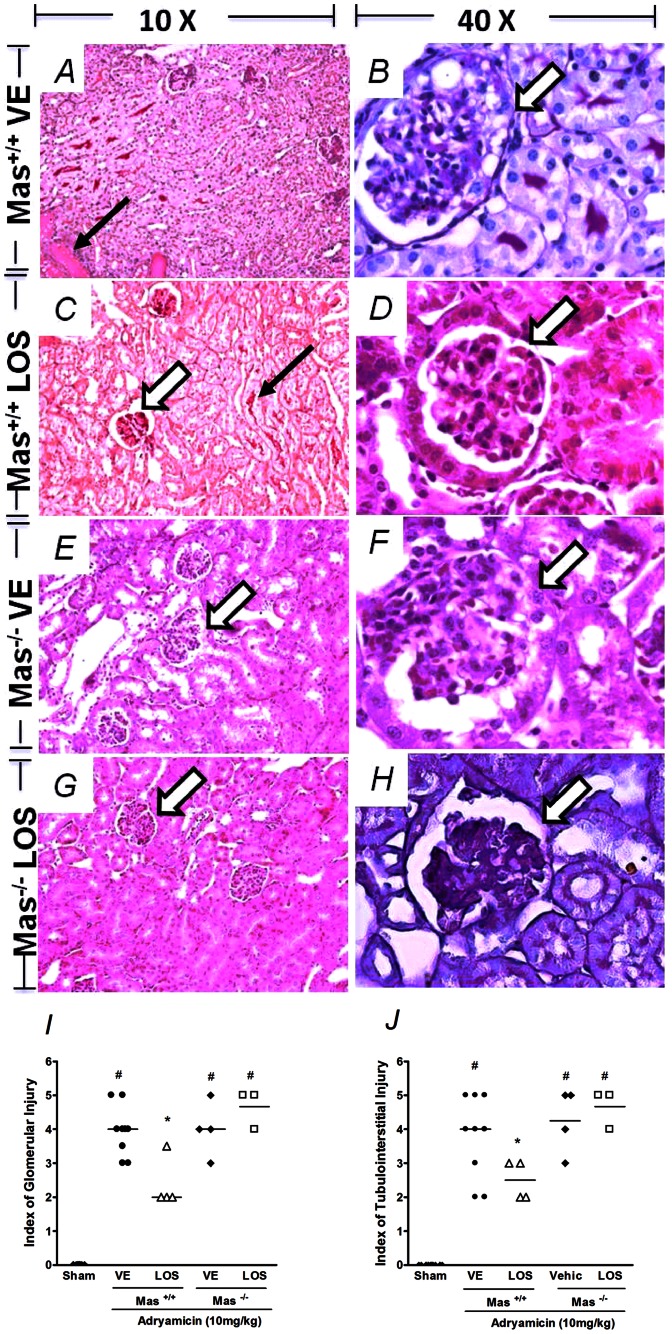
Treatment with losartan protects from adriamycin-induced renal damage in wild type (Mas^+/+^) but not Mas deficient (Mas^−/−^) mice. Adriamycin (10 mg/kg) was injected in the tail vein of Mas^+/+^ (A–D) and Mas^−/−^ (E–H) FVBN mice. Animals were treated with water (VE, A–B and E–F) or Losartan (LOS, 10 mg/kg, C–D and G–H) and morpohological changes evaluated at day 14 after adriamycin injection. Glomerular (large arrow) and tubular injuries (thin arrows) are showed. Indices of tubulointerstitial (I) and glomerular (J) injuries were graded in a blind manner, as described in the Methods section. PAS-stained sections and magnification 10× (A, C, E, G) and 40× (B, D, F, H). Symbols represent results in single animals and the trace is median value. (*) for P<0.05 when compared to VE-treated group and (#) for P<0.05 when compared to sham group.

Mice with genetic deletion of Mas receptor in FVB/N background (*Mas* ^−/−^) were given ADR in order to evaluate the role of endogenous Mas receptor in this model of nephropathy. As displayed in Figure 5 (A–B and E–F), histological changes of ADR-induced nephropathy were similar in *Mas* ^−/−^ and wild type mice (*Mas* ^+/+^). To evaluate the contribution of Mas receptor for the renoprotective actions caused by the treatment with the AT_1_ antagonist Losartan, a dose of 10 mg/Kg of this medication was also given to *Mas* ^−/−^ and *Mas* ^+/+^ mice with ADR-induced nephropathy. The treatment with Losartan was able to reduce renal histological injury indexes only in *Mas^+/+^*, but not in *Mas* ^−/−^ mice (Figure 5C–D and 5G–H). Scores for glomerular and tubule-interstitial damage are shown in Figures 5I and 5J, respectively.

## Discussion

Major findings of the present study can be summarized as follows: (i) treatment with AVE 0991, an orally-active Mas receptor agonist, significantly improved renal function parameters, reduced urinary protein loss and attenuated histological changes in a murine model of ADR-induced nephropathy. (ii) Renoprotective actions of AVE 0991 were very similar to those produced by the administration of Losartan, an AT_1_ receptor antagonist. (iii) Renoprotection induced by AVE 0991 was associated with reduction in urinary levels of the fibrogenic cytokine, TGFβ1. (iv) In ADR-induced nephropathy, mRNA expression for both angiotensin receptors, AT_1_ and Mas, were decreased. On the other hand, the treatment with Losartan significantly increased the mRNA expression for Mas receptor and for ACE2 in renal tissue. (v) Finally, renoprotective effects of Losartan were blunted in mice with genetic deletion of Mas receptor, demonstrating that Mas receptor activation is essential for the renoprotective effects of AT_1_ receptor antagonists.

ADR-induced nephropathy in mice mimics several aspects of the renal dysfunction observed in human nephrotic syndrome [23]. It is considered to be an experimental model of focal segmental glomerulosclerosis, which is characterized by interstitial infiltration, glomerular fibrosis, and proteinuria [18], [19], [24]. Accordingly, we have found increased urinary protein excretion, reduced serum albumin, mild elevation in systolic blood pressure and significant histological changes in glomerular and tubular compartments, as it has been reported in other studies [19], [25]–[27].

Our study represents the first evidence of renoprotection obtained with Mas receptor activation by oral administration of its agonist, AVE 0991, in ADR-induced nephropathy. Studies using mice with genetic deletion of the Mas receptor showed controversial results in relation to renal function and renal histopathology in different models [16], [17]. While Pinheiro et al (2009) [16] showed that the genetic deletion of Mas receptor in C57Bl/6 background mice led to glomerular hyperfiltration, proteinuria and renal fibrosis, Esteban et al (2009) [17] reported that renal deficiency of Mas diminished renal damage in unilateral ureteral obstruction and ischemia/reperfusion injury, and that the infusion of Ang-(1–7) to wild-type mice elicited an inflammatory response. Furthermore, animal models of renal diseases have also shown discrepant findings [26], [27]. Zhang et al [27] showed that a 5-day infusion of Ang-(1–7) reduced proteinuria and improved glomerulosclerosis in a rat model of thy-1 induced glomerulonephritis, whereas van der Wouden et al reported that infusion of Ang-(1–7) was not able to reduce proteinuria in ADR-induced nephropathy [26]. Velkoska et al (2011) showed that a 10 day infusion of Ang-(1–7) in rats with subtotal nephrectomy was associated with deleterious effects on blood pressure and the heart, with increase in cardiac ACE, and decrease in cardiac ACE2 activity [28]. Dilauro et al (2010) suggested a renoprotective action for ACE2 activation, while no effect was obtained following Ang-(1–7) infusion in mice subjected to subtotal nephrectomy [25]. Consequently, the effects of Ang-(1–7) in the kidney appear to be importantly influenced by experimental conditions and the previous level of RAS activation. Indeed, differences between species, local and systemic concentrations of Ang-(1–7), nephron segment, level of RAS activation and sodium and water status can be responsible for these divergent effects on renal function [25], [26], [28]. In this study, we have clearly shown renoprotective effects of AVE0991 in a murine model of ADR-induced nephropathy. Oral administration of this Mas receptor agonist significantly improved renal function parameters, reduced urinary protein loss and protected against renal tissue damage.

Immunohistochemical data have shown a similar distribution for Ang-(1–7), ACE2 and Mas within the kidney [29], placing the key components together for activation and activity. Mas receptor was detected at different nephron segments such as the juxtaglomerular apparatus, proximal tubules, and collecting ducts of mice [16] and at both cortical and medullar regions of rat kidneys [30]. Consistent with the latter finding, it has been shown that the biological effects of Ang-(1–7) in the kidney are primarily mediated by Mas [7], [8], [16]. Mas-deficient mice have fluid retention, glomerular hyperfiltration, microalbuminuria, increased collagen deposition and mRNA overexpression of AT_1_ receptor and TGF-β in renal tissues [16]. These results indicate that the lack of Mas may lead to RAS imbalance with unopposed actions of the ACE/Ang II/AT_1_ axis in the kidney.

Although several studies demonstrate divergent roles to endogenous Mas receptor activation on progression of the renal disease (Pinheiro and Esteban, for example) [16], [17], the present study demonstrated another important finding: the absence of Mas receptor did not affect ADR-induced injury, suggesting that the role of endogenous activation of Mas receptor is not as relevant as the beneficial effects of exogenous stimulation by AVE 0991 administration. Recently, we demonstrated similar effect in a model of renal ischemia and reperfusion, in which *Mas* KO mice presented similar levels of creatinine and of renal neutrophil influx when compared to wild type mice [31]. We have also shown a minor role for Mas deficiency in worsening the injury observed in an antigen-induced-arthritis mice model (AIA), while pharmacological activation of Mas receptor had meaningful biological effects and efficiently controlled articular inflammation in the same experimental model [32]. Despite the absence of an endogenous role for Mas receptor, it is clear that the exogenous activation of this receptor provided important renoprotective effects in the context of ADR-induced nephropathy.

The pathways by which AVE 0991 reduced proteinuria and attenuated renal tissue injury were not fully elucidated. The detection of proteinuria in ADR-induced animals suggests direct podocyte injury. Previous studies also support the direct role of ARBs in renal podocytes [33]–[35]. Matsusaka et al (2010) showed that ARBs attenuated podocyte injury, proteinuria, and glomerulosclerosis in the NEP25 model [33]. The podocyte protection was independent of the local inhibition of AT_1_ receptors. Naito et al (2010) showed, in a model of 5/6 nephrectomy, that the podocyte protection following ARBs treatment was due not only to the blockade of AT_1_ receptor, but also to Ang II effects mediated by AT_2_ receptor [34]. More recently, Shimizu et al (2012) also showed that ARB exerts podocyte protection in a mice model of HIV-1 nephropathy. In the present study, we did not evaluate the direct effect of Losartan or AVE 0991 on podocytes [35]. However, the treatment with both Losartan and AVE0991 reduced urinary protein loss and glomerulosclerosis.

Another important pathway elicited by Losartan and AVE 0991 treatment was the reduction of renal levels of TGF-β in the present experimental model. TGF-β expression in the kidney is thought to be a final common pathway leading to the development of structural damage and fibrosis in a range of glomerular diseases [36], [37]. This cytokine can be synthesized by numerous cells including macrophages, T and B lymphocytes fibroblasts, and resident renal cells. By binding to AT_1_ receptor, Ang II may promote progression of renal fibrosis via the production of TGF-β1 [38]. In this regard, Crowley et al (2009) showed that the administration of the AT_1_ receptor antagonist, Losartan, significantly reduced the mRNA expression for TGF-β in renal tissue, whereas animals with genetic deletion of this receptor exhibited an increased expression of the cytokine [39]. Accordingly, our study also showed that Losartan reduced renal tissue levels of TFG-β in ADR-induced nephropathy.

Recent studies indicated that the activation of ACE2-Ang-(1–7)-Mas receptor axis might attenuate fibrogenic processes by decreasing TGF-β levels or expression in many tissues [40]–[45]. For instance, in variours models of myocardial hypertrophy, the administration of Ang-(1–7) or AVE 0991 reduced local levels of TGF-β and produced cardiac remodeling [41]. Once again, studies have shown divergent results concerning renal tissue. Consistent with our findings, it was previously shown that Ang-(1–7) decreased TGF-β levels in rat proximal tubular cells [44] and reduced renal fibrosis in experimental diabetic nephropathy [45]. Su et al (2006) showed that Ang-(1–7) inhibited Ang II-stimulated phosphorylation of ERK1/2, p38 MAPKs and c-Jun N-terminal kinase in culture rat proximal tubular cells, an effect reversed by pre-treatment with A-779. Ang-(1–7) also prevented Ang II-induced production of TGF-β1 in proximal tubular cells. Thus, the generation of Ang-(1–7) by proximal tubular ACE2 could counteract the proliferative effects of locally produced Ang II [42]. In addition, the genetic deletion of Mas receptor led to high mRNA expression of TGF-β in renal tissue [16]. In contrast, other authors reported that Ang-(1–7) increased TGF-β in human renal mesangial cells (56) and accelerated the progression of experimental diabetic nephropathy [44], [45]. Although these findings are conflicting, cell-specific signaling pathways associated with Ang-(1–7) in the kidney could play a role in the variable response. In this study, we have detected a decrease in renal TGF-β levels elicited by the Mas receptor agonist administration. Our data support an anti-fibrogenic role for ACE2-Ang-(1–7)-Mas receptor axis at renal tissue, as previously demonstrated for heart [46] and liver tissues [47].

AT_1_ receptor antagonism is considered a first-line strategy to control the progression of chronic kidney diseases [3], [6]. For this reason, the renal effects of AVE 0991 administration were compared with those related to the treatment with Losartan, an AT_1_ receptor antagonist. It was noteworthy that AVE0991 and Losartan reduced histological injury indexes, urinary protein and TGF-β levels in the same way. The renoprotective actions of ARBs clearly involve multiple pathways including anti-proliferative and anti-fibrogenic effects [3], [6], [48], [49]. In particular, an altered balance between Ang II and Ang-(1–7) might be related to the mechanism of action of AT_1_ receptor blockade, since this treatment increased the circulating levels of Ang-(1–7) [50], [51]. In addition, Kostenis et al (2008) suggested that the Mas receptor is a physiologic antagonist of the AT_1_ receptor [52].

Therefore, we evaluated whether the presence of Mas receptor was relevant for the described protective effects of the treatment with AT_1_ receptor blockers by using mice with genetic deletion of Mas receptor (Mas^−/−^). Mas^−/−^ animals exhibited the same degree of histological injury when compared to wild type mice (Mas^+/+^). On the other hand, the treatment with Losartan was able to attenuate renal injury only in Mas^+/+^ mice, but not in Mas^−/−^ animals. It would have been interesting to examine whether, in Mas knockout mice, AVE 0991 compound could improve or not ADR-induced renal damage. Previous studies of our group showed that AVE 0991 was not able to affect renal function parameters [16] or leukocyte infiltration in experimental arthritis [15] in Mas KO animals. In addition, the binding of AVE 0991 to renal tissue was absent in Mas KO animals [16]. Therefore, based on the selectivity of the compound and previous results of our group, we do believe that the presence of Mas receptor is critical for the renoprotective effects of AVE 0991.

It should also be considered that this model of nephropathy produced significant reduction in the mRNA expression for both angiotensin receptors, Mas and AT_1_, but the treatment with Losartan increased only the expression of Mas receptor, without changing mRNA levels for AT_1_ receptor. Moreover, Losartan also increased renal expression of ACE2, the main responsible for Ang-(1–7) synthesis at renal tissue [53]. Corroborating the present data, previous studies reported similar effects of ARBs on the modulation of ACE2Ang-(1–7)/Mas receptor axis at different sites [54]–[58]. In this regard, Ferrario et al (2005) showed that Losartan increases plasma and urinary levels of Ang-(1–7) and renal ACE2 activity, without changing the expression of Mas receptor, AT_1_ or ACE in Lewis rats. Igase et al (2011) in a model of hypertensive nephropathy showed that olmesartan treatment increased plasma levels of Ang-(1–7) leading to cardiprotective and renoprotective effects [55]. In a model of ADR-induced heart failure in Male Sprague-Dawley rats, Zong et al (2011) detected a decrease of plasma Ang-(1–7) levels and reduced myocardial expression of Mas receptor, while the treatment with telmisartan or losartan increased Ang-(1–7) levels and suppressed myocardial AT_1_ receptor expression without changing the expression of Mas [58]. Recent studies of Sukumaran et al (2011 and 2012) showed that the protein and mRNA levels of Mas receptor, ACE2 and Ang-(1–7) were upregulated in olmesartan treated group in experimental autoimmune myocarditis and these changes in RAS components decreased the expression of inflammatory markers [56], [57]. Taken together, these findings indicated that ACE2/Ang-(1–7)/Mas receptor axis activation participate in the renoprotection triggered by ARB

In conclusion, this study shows that the Mas receptor agonist, AVE0991, has renoprotective actions in ADR-induced nephropathy. The effects of AVE0991 were comparable to those of the AT_1_ receptor antagonist, Losartan. Beneficial effects of AVE0991 and of Losartan were related to the reduction of urinary levels of the fibrogenic cytokine, TGF-β. Furthermore, the presence of Mas receptor seemed to be critical for the renoprotective actions of AT_1_ antagonists. Further research on the contribution of the ACE2/Ang-(1–7)/Mas axis to renal pathophysiology should lead to the development of new pharmacological approaches for human nephropathies.
